# The role of bacteria in cancer therapy – enemies in the past, but allies at present

**DOI:** 10.1186/s13027-018-0180-y

**Published:** 2018-03-15

**Authors:** Shiyu Song, Miza S. Vuai, Mintao Zhong

**Affiliations:** 1 Department of Medical Microbiology, Dalian Medical University, 9 Western Section, Lvshun South Road, Lvshunkou District, Dalian, 116044 China; 2 Department of Natural Science, State University of Zanzibar (SUZA), P.O Box 146, Zanzibar, Tanzania

**Keywords:** Cancer, Bacteria, Detection, Therapy

## Abstract

In recent decades, bacteria’s therapeutic role has aroused attention in medicinal and pharmaceutical research. While bacteria are considered among the primary agents for causing cancer, recent research has shown intriguing results suggesting that bacteria can be effective agents for cancer treatment – they are the perfect vessels for targeted cancer therapy. Several bacterial strains/species have been discovered to possess inherent oncolytic potentials to invade and colonize solid tumors in vivo. The therapeutic strategy of using bacteria for treating cancer is considered to be effective; however, the severe side effects encountered during the treatment resulted in the abandonment of the therapy. State-of-the-art genetic engineering has been recently applied to bacteria therapy and resulted in a greater efficacy with minimum side effects. In addition, the anti-cancer potential of tumor-targeting bacteria through oral administration circumvents the use of the intravenous route and the associated adverse effects. This review aims to provide a comprehensive summary of the latest literature on the role of bacteria in cancer treatment.

## Background

Despite the advancement in cancer treatment and detection, cancer has remained a major health problem and one of the leading causes of deaths worldwide. About 600,000 cancer deaths were projected to occur in the United States in 2016, out of 1.6 million newly reported cancer cases [1]. Similarly, about 3million cancer deaths were estimated to occur in China in 2015, out of 4 million reported cancer cases [2].

The conventional chemotherapeutic agents used for the treatment of cancer possess non-specific toxicity toward normal body cells. Also the body cells that are exposed to chemotherapy often become resistant to drugs because of enhanced capability to repair DNA defects in cellular machinery which intervenes apoptosis. The exposed cells increase the production of enzymes that cause detoxification of drug and drug delivery services [3]. These inherent complications of chemotherapy, which include drug resistive mechanism of cells caused by chemotherapy, have caused scientists to focus on examining the potential of using bacteria and their compounds for anti-cancer therapy [3].

**Fig. 1:**
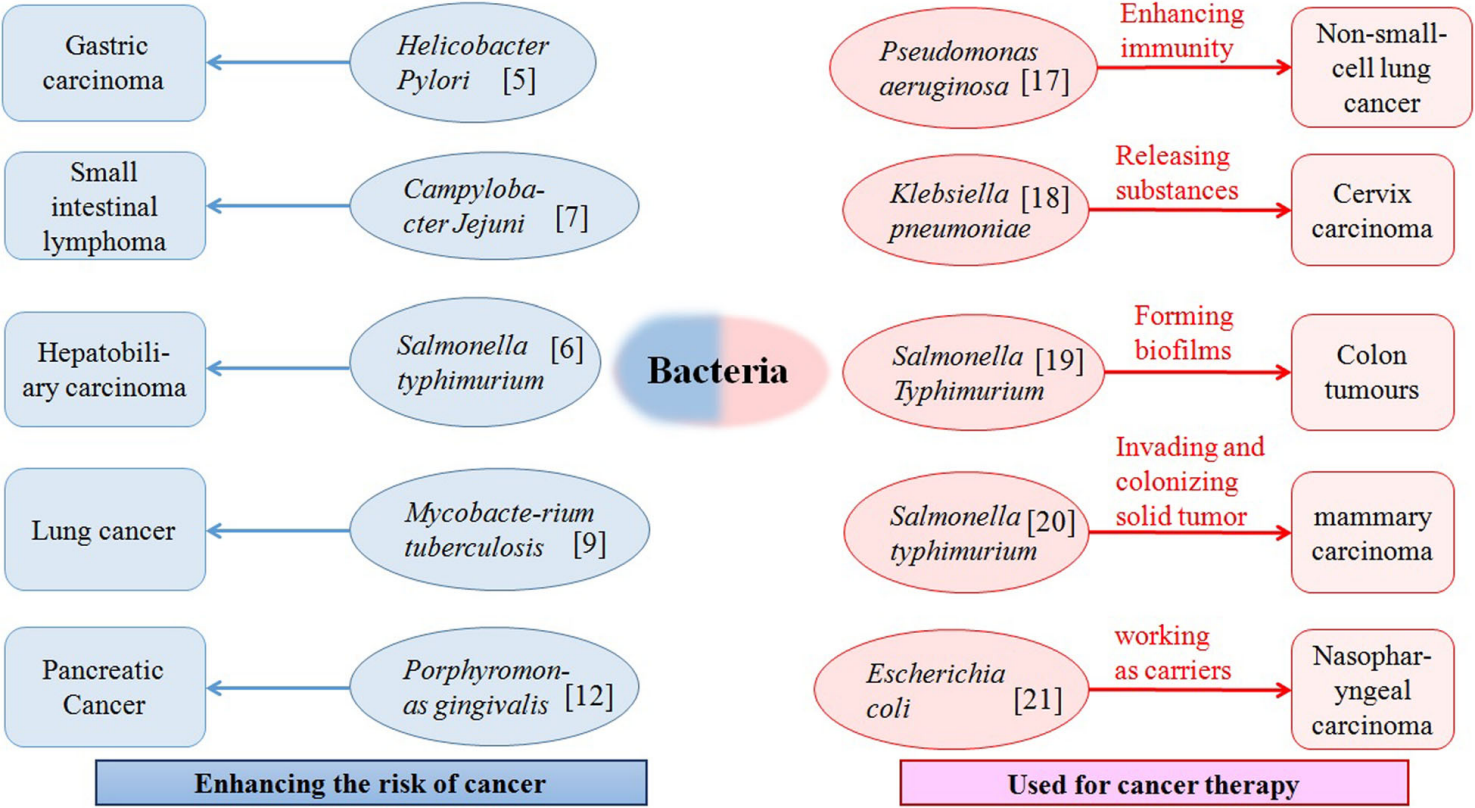
The schematic presentation for Bacteria strains and their double roles as cancer causing agents and cancer therapeutic agents

Bacteria are carcinogens and tumor promoters [4]. Bacteria produce toxins that disrupt the cellular signal thus perturbing the regulation of cell growth. Also, they are potential tumor promoters through inducing inflammation. Some bacteria strains notable for causing cancer are presented in Fig. 1. The strains include *Helicobacter pylori,* which is associated with gastric cancer [5], *Salmonella typhi* which is associated with hepatobiliary carcinoma [6], *Campylobacter Jejuni* which is associated with small intestinal lymphomas [7], *Chlamydia psittaci* which is associated with ocular lymphomas [8], *Mycobacte-rium tuberculosis* which is associated with lung cancer [9], and *Citrobacter rodentium,* which is associated with human colorectal cancer [10]. In addition, the enzymes produceed by bacteria are potential carcinogens, such as peptidyl arginine deaminase (PAD) enzymes that are found in oral bacteria and associated with pancreatic cancer [11, 12]. Also, quorum sensing peptides, such as PhrG from *Bacillus subtilis*, competence stimulating peptide (CSP) from *Streptococcus mitis* and extracellular death factors (EDF) from *Escherichia coli* together with their tripeptide analogue, are reported to promote tumor cell invasion and angiogenesis through type I collagen extracellular matrix, which influences tumor metastasis [13]. The quorum sensing peptides down-regulate microRNA-222 and initiate angiogenesis which promotes neovascularization and results in tumor metastasis [14].

Conversely, bacteria have shown great potential for cancer therapy. Bacteria of many species demonstrate the surprising ability to invade and colonize solid tumors, which often results in neoplasm growth retardation, and in some instances, complete tumor clearance [15]. Different strains of *Clostridia, Bifidobacteria* and *Salmonella* are capable of colonizing the hypoxic area of the tumor and destroy the tumor cells. Therefore, they are potential strains for selective tumor targeting therapy [16–21] (Fig. 1).

Bacteria create anti-tumor effects through the depletion of nutrients required for cancer cell metabolism [22]. The tumor tissues that are deoxygenated nurture the accumulation of obligate anaerobic bacteria – which only survive in the anoxic region [23]. Observation has shown that the systemic administration of *Salmonella* bacteria flushed into the solid tumor through severe haemorrhaging area, the area which leads to necrotic regions in which bacteria proliferate [24], colonized the tumor and decreased the proliferation of the tumor. The necrotic regions are formed because of the reduction of oxygen and nutrient supply, which leads to the breaking down of blood vessels in the hemorrhagic area. This causes the tumor cells in the center of the tumor to die from starvation and suffocation [24]. The tumor microenvironment may be conducive to bacterial survival and growth, as it may provide protection from the host immune system and nutrients [25].

Bacteria mediated tumor therapy (BMTT) has been demonstrated for centuries. However, the associated adverse side effects hinder its development. Adequate balance between the control of infection and the therapeutic benefit of bacteria is an essential requirement for a successful BMTT, but this can only be achieved via the heat-inactivation method [26]. Recently, state-of-art genetic engineering has increased the ability to alter bacterial strains [27]. Such alteration reduces bacteria side effects while increasing their therapeutic benefits. For example, the well-established bacillus Calmette–Guerin (BCG) vaccine for the treatment of human bladder cancer is arguably superior to intravesical chemotherapy for superficial disease. The therapy is commonly used as the first-line adjuvant treatment [28]. Similarly, tumor-detecting bacteria provide a sensitive and minimally invasive method to detect tumor recurrence, monitor treatment efficacy, and identify the onset of metastatic disease [29]. For example, probiotic *Escherichia coli* Nissle 1917 has been used to develop the orally administered diagnosis that can noninvasively indicate the presence of liver metastasis by producing easily detectable signals in urine [30]. Such genetic engineering approaches have paved the way for further development of promising bacteria-based cancer therapy.

## Bacteria as anti-cancer agents through enhancing human immunity

An important factor that applies to the spontaneous regression of cancer is the duality of the immune system [31]. Bacteria interact with the host as either pathogen or normal flora. The pathogenic interaction of bacteria enhances the immune system of the host in different ways.

### Activating inflammasome pathways

The ∆ppGpp *Salmonella typhimurium* strain activates inflammasome pathways by damaging the signals released from cancer cells. This phenomenon significantly increases the amount of inflammatory cytokine IL-1β, TNF-α and Il-18 in tumors, which results in drastic tumor growth suppression [32]. IL-1β is the proinflammatory cytokine that plays a pivotal role in immunity against pathogens [33]. The IL-1β is secreted by LPS (lipopolysaccharides) during the activation of toll like receptor (TLR4) and inflammasomes, which then causes the damage to cancer cells [32]. The cancer cells are also damaged when bacteria activate inflammasomes in BMDM, which is involved in phagocytosis of damaged cancer cells by macrophages [32]. Therefore, the ∆ppGpp *Salmonella typhimurium* shows therapeutic efficacy for cancer through involving the inflammasome pathway [32].

### CD4, CD25 and CD8 anti-tumor effectors T cell responses

Anaerobic bacteria such as *E. Coli*, which are capable of engulfing the solid tumors, are indirectly involved in clearance of some tumor cells (e.g. CT26) through infectious-defense mechanism. Once these bacteria invade the host, they stimulate the initiation of the defense mechanism of the host, which results in the production of lymphocytes T cells. The produced lymphocytes T cells are significantly involved in anti-tumor activity. During the induction phase of bacterial infection, CD8^+^ T cells are the only effectors responsible for tumor clearance; whereas, in the memory phase the clearance also involves CD8^+^ and CD4^+^T cells [34]. CD8^+^ T is reported to take part in the clearance of the original tumor after bacterial infection [34]. Similarly, the anti-tumor effectors T cells (CD4^+^ and CD8^+^) have the potential to block the formation of a new set of tumors. The CD8^+^ T cell is said to have the additional ability to eradicate even the already established tumors [34]. Furthermore, the lymphocytes (CD4 and CD25) and cytokines are reported to be the novel therapies for colon cancer in humans [35]. Their role in host immune response on carcinogenesis is invaluable [34, 35]. The regulatory cells (like CD4 and CD25) are capable of reducing the severity of inflammatory bowels and lower the risk of colon cancer [35]. In addition, the introduction of regulatory cells into chronically infected mice with established cancer showed the reduction of the severity of colitis, epithelial dysplasia, and cancer [35].

### The TNF-α innate immune system in bacteria-based tumor necrosis

The tumor necrosis factor (TNF- α) has the potential to damnify the vascular endothelial cells. TNF-α plays the significant part in the formation of the large haemorrhaging area that appears within the tumor. Systematic administering of *Salmonella enterica serovar Typhimurium* to mice models indicated that haemorrhaging increases the flushing of bacteria into the solid tumor resulting in necrosis [24]. Moreover, the activeness of the innate immune system of the host, especially neutrophilic granulocytes, is proportional to the area of necrotic. Neutrophiles function to separate the bacteria-containing necrotic region from the bacteria that migrate into the tumor from viable tumor cells. The depletion of host neutrophils increases the number of bacteria in the tumor and increases the ability of bacteria to migrate into vital tumor tissue [36]. Thus, the complete eradication of the established tumors could be attained with the increasing size of necrosis. Similarly, the depletion of host neutrophils amplifies the bacteria-mediated tumor therapy.

## Bacteria as anti-cancer agents through released substances

Some substances secreted by bacteria, such as enzymes, can inhibit the growth of tumors. Several experimental studies discovered the therapeutic potential of different substances released from bacteria for treating cancer cell lines.

### Bacteriocins

Bacteriocins are cationic peptides that are synthesized by almost all groups of bacteria ribosomally. Bacteriocins are non-immunogenic, biodegradable and contain cancer cell-specific toxicities. The bacteriocins have the potential to serve as synergistic agents to conventional cancer drugs [37]. Cancer cell membranes predominantly carry negative charge; thus bacteriocins preferentially bind to cancer cell membranes than to the normal cell membranes, which are neutral in charge and selective for binding of bacteria [38].

Colicins, the bacteriocin secreted from Enterobacteriaceae such as *Escherichia coli* (*E.coli)*, are known to have anti-cancer activities against a variety of human tumor cell lines in vitro, including breast cancer, colon cancer, bone cancer and uteri cell line HeLa (human cervical adenocarcinoma) [38]. Microcin E492, part of Colicins from *Klebsiella pneumoniae*, was found to induce apoptosis in some human malignant cell lines such as HeLa, Jurkat (T cell derived from acute T cell leukemia), RJ2.25 (a variant of Burkitt’s lymphoma), and colorectal carcinoma cells, with no effect on normal cells [17]. Pediocin isolated from *Pediococcus acidilactici* K2a2-3 was reported to have cytotoxic activities against HT29 (human colon adenocarcinoma) and HeLa cell lines [39]. Similarly, Nisin (the bacteriocins from *Lactobacillus lactis*) possesses cytotoxic effect on MCF-7 (human breast adenocarcinoma cell line) [40], HepG2 (liver hepatocellular carcinoma) [41], and HNSCC (head and neck squamous cell carcinoma) [42], both in vitro and in vivo. Conversely, Nisin is non-toxic and safe to humans, WHO has approved it for human consumption.

Furthermore, partially purified bacteriocins produced by certain bacteria such as *Pseudomonas aeruginosa* have shown anti-cancer activities [43]. Pyocin, the bacteriocin produced by more than 90% of *Pseudomonas aeruginosa* strains [44–46], showed lethal effect on the L6OT mice fibroblast cell line [47]. Likewise, purified and partially purified pyocin S2 showed the cytotoxicity effect on tumor cell line HepG2 and Im9 (Human immunoglobulin-secreting cell line derived from multiple myeloma) with no effect on normal cell line HFFF (Human fetal foreskin fibroblast) [48].

### Phenazine 1,6-di-carboxylic acid (PDC)

Multiple phenazine metabolites such as phenazine 1-carboxylic acid (PCA) and Phenazine 1,6-di-carboxylic acid (PDC) are derived from bacteria strains (e.g. *Pseudomonus aeruginosa*). The PDC phenazine was first isolated from *Streptomyces* species; it was demonstrated to be the potential agent for controlling metabolism and biofilm formation in *Candida albicans* [49, 50]. Compared to other phenazine metabolites, PDC showed a substantially broader spectrum of cytotoxicity effect towards a number of cancer cells of different origins, including HT29, HeLa, and MCF7 cell lines, with less activity on DU145 (Human prostate cancer cell lines) [51].

## Bacteria as anti-cancer agents through biofilms

Biofilm is a primitive form of multicellular life that provides bacteria with tolerance strength against antibiotics and host defense mechanisms [52]. Biofilms are common to opportunistic bacterial pathogens such as *Salmonella tyhimurium*, and they (the biofilms) are decisive in the pathogenesis of chronic infectious diseases [53]. *Salmonella tyhimurium* and some other pathogens are known to cause severe haemorrhage within the tumor. Once the haemorrhae is activated, it induces the production of T cells that are very significant in biofilm induction [53]. Notwithstanding the etiopathogenesis of biofilms and its protective role that allows bacteria to escape from the host defense system [53], recent discoveries have revealed the potential ability and efficacy of biofilms in cancer therapy.

Anti-cancer drugs cause the induction of biofilm formation during cancer treatment, which results in metastasis distraction [54, 55]. Similarly, the formation of bacteria biofilm on cancer cells during the SOS response results in metastasis disruption. Thus bacteria biofilm shows potential usefulness in cancer treatment [56]. Bacteria biofilm can affect colon cancer development and progression through modifying cancer metabolome to produce a regulator of cellular proliferation [56]. Also, the bacterial macromolecules necessary for biofilm formation such as proteins and DNA coat cancer cells to block metastasis [57]. For example, polysaccharides released by *Streptococcus agalactiae* inhibit adhesion of cancer cells to endothelial cells, an essential step in cancer metastasis [58]. Furthermore, [59] demonstrates the potential application of iron oxide nanowires from a biofilm waste produced by bacteria (*Mariprofundus ferroxydans*) as a new multifunctional drug carrier for cancer therapy and cancer hyperthmia.

While the above hypotheses avow for the potential of bacterial biofilm in cancer therapy, the evidence is relatively insufficient to build-up the case. However, the efficacy of bacteria biofilm for metastasis distraction calls for the further examination and investigation of the anti-cancer ability of bacteria biofilm.

## Bacteria as a carrier for cancer therapeutic agents

Apart from their direct anti-cancer effect, tumor-targeting bacteria can also be used as carriers for cancer therapeutic agents in cancer treatments. Recent studies have revealed that bacteria are capable of targeting both primary tumors and metastasis [60–63].

### Bacteria-mediated anti-angiogenesis therapy

The growth and metastasis of solid tumors depend on the formation of new blood vessels (angiogenesis). Thus blocking tumor angiogenesis can be a reasonable approach to treat solid tumors. Jia et al. [64] applied the combination therapy of a low dose of *Salmonella* (attenuated, auxotrophic) and rhEndostatin in a murine model of malignant melanoma which resulted in the reduction of the tumor growth. The therapy is argued to be safer and effective, but also economically desirable – it decreases possible side effects and lowers the therapeutic expense. Moreover, Li et al. [65] used *Bifidobacterium adolescentis* (non-pathogenic) as a vector for the expression of endostatin within tumors. Their findings showed that *Bifidobacterium adolescentis* strongly inhibit the angiogenesis and significantly inhibit local tumor growth. *Bifidobacterium longum* efficiently delivered the anti-angiogenic protein (endostatin) to murine liver tumors and induced anti-tumor activity [66, 67]. In addition, the oral anti-angiogenic bacterial vaccines directed against vesicular endothelial growth factor receptor 2 (VEGFR-2) were proven efficacious in animal models of malignant melanoma, colorectal carcinoma and lung cancer [68].

The combined therapy of bacteriolytic and anti-angiogenic using tumor-targeting bacteria has also shown promising results. Bacteria are capable of invading the poorly perfuse tumor areas (which are not accessible by systemically administered agents) using their unique metabolic features. They cause the inhibition of angiogenisis to kill residual tumor cells, and hence significantly increase the chance for tumor eradication [69]. In addition, the anti-tumor effect can be enhanced through co-administration of tumor necrosis factor-related apoptosis-inducing ligand (TRAIL) and endostatin [70].

### The combined treatment of bacteria and viruses

Oncolytic viruses (OVs) have shown positive outcomes for cancer treatment through their tumor-selective replication and multi-modality attack against cancers [71]. The viral-mediated oncolysis cancer therapy approach is potentially more effective and less toxic than current treatment regimes. While bacteria are arguably better at targeting the tumor, viruses are argued to possess unprecedented abilities to kill cancer cells. Apart from the generic effective killing mechanisms, certain virus mutants have the ability to selectively kill cancer cells [72]. Bacteria have the ability to disseminate the virus inside the tumor and induce a strong immune response against tumor antigens [73]. In their experiment, Cronin et al. [74] found that non-pathogenic bacteria (*Escherichia coli*) expressing B18R enhanced the oncolytic potential of the vesicular stomatitis virus (VSVΔ51) to reduce tumor growth, and thus prolonged the survival of an aggressive tumor model.

### Bacteria-based microrobot (Bacteriobot)

The *bacteriorobot* technique is a new innovative theranostic methodology of bacteria-based fabrication for tumor therapy. The technique uses bacteria as microactuators and microsensors to deliver microstructures for targeting and treating solid tumors [75]. Various types of biomedical microrobots have been invented through the convergence of technologies from micro electromechanical system (MEMS) with nano- and bio-technologies [76, 77]. In an attempt to develop microrobot therapy, Park et al. [75] encapsulated therapeutic bacteria (*Salmonella typhimurium*) in biocompatible/biodegradable alginate microbeads and attached flagellated bacteria (*Salmonella typhimurium*) on the microbead to fabricate a bacteria-based microrobot in the targeted tumor region. The bacteria-encapsulated delivery system protects the bacteria from being attacked by the immune system, which is safer than the direct inoculation of bacteria [78].

## Conclusion

Bacteria are the double-edged sword in cancer therapy. Using bacteria for cancer therapy is feasible and its potential to treat solid tumors has been known for decades. However, the clinical application of this therapy never became routine because of the adverse uncontrollable side effects. In an effort to overcome these side effects, some attenuated species of bacteria capable of treating cancer have been recently identified and studied. These species of bacteria are considered safe for cancer therapeutic application with little or no side effects. While bacteria alone may not demonstrate fully therapeutic potential, their modifications as anti-tumor agents, anti-oncogenes or immunogenic antigens, and their combination with other therapeutic processes will improve their potential for cancer therapy. The arena of using bacteria as an anti-cancer agent is still new; further studies are imperative to scrutinize the clinical significance of bacteria-based cancer therapy. The findings presented in this review suggest that this promising cancer therapy needs to be optimized and developed further.

## Abbreviations

BCG: Bacillus Calmette–Guerin
BMDM: Bone marrow derived macrophages
BMTT: Bacteria mediated tumor therapy
CT26: Murine colon carcinoma
DU145: Human prostate cancer cell lines
HeLa: Human cervical adenocarcinoma
HepG2: Liver hepatocellular carcinoma
HFFF: Human fetal foreskin fibroblast
HNSCC: Head and neck squamous cell carcinoma
HT29: Human colon adenocarcinoma
Jurkat: T cell derived from acute T cell leukemia
L6OT: Mice fibroblast cell line
LPS: lipopolysaccharides
MCF7 cells: Human breast adenocarcinoma cell line
MEMS: Micro Electro Mechanical System
NSCLC: Non Small Cell Lung Cancer
OVs: Oncolytic viruses
PAD: Peptidyl arginine deaminase
PAP: *Pseudomonas aeruginosa* preparation
PCA: Phenazine 1-carboxylic acid
PDC: Phenazine 1,6-di-carboxylic acid
RJ2.25: Variant of Burkitt’s lymphoma
TLR4: Toll like receptor
TNF- α: Tumor necrosis factor
TRAIL: Tumor necrosis factor-related apoptosis-inducing ligand
VEGFR-2: Vesicular endothelial growth factor receptor 2
VSVΔ51: Vesicular Stomatitis Virus
